# Supplementary material for: Pathway of Hsp70 interactions at the ribosome
Source: Nat Commun. 2021 Sep 27;12:5666. doi: 10.1038/s41467-021-25930-8 (PMC8476630; doi:10.1038/s41467-021-25930-8)
Supplement: Supplementary file 1 — Supplementary Information [file 41467_2021_25930_MOESM1_ESM.pdf]

Supplementary Information:

Pathway of Hsp70 interactions at the ribosome

Kanghyun Lee[1,4], Thomas Ziegelhoffer[1,4], Wojciech Delewski[1],
Scott E. Berger[1,2], Grzegorz Sabat[3], and Elizabeth A. Craig[1,*]

[1]Department of Biochemistry, University of Wisconsin-Madison, Madison Wisconsin, 53706, USA
[2] Department of Chemistry, Lafayette College, Easton, PA 18042, USA
[3]Biotechnology Center, University of Wisconsin-Madison, Madison Wisconsin, 53706, USA

[4]These authors contributed equally to this work.

*Correspondence should be addressed to E.A.C. (ecraig@wisc.edu)

Supplementary Information includes 6 figures and 3 tables

Supplementary Figures

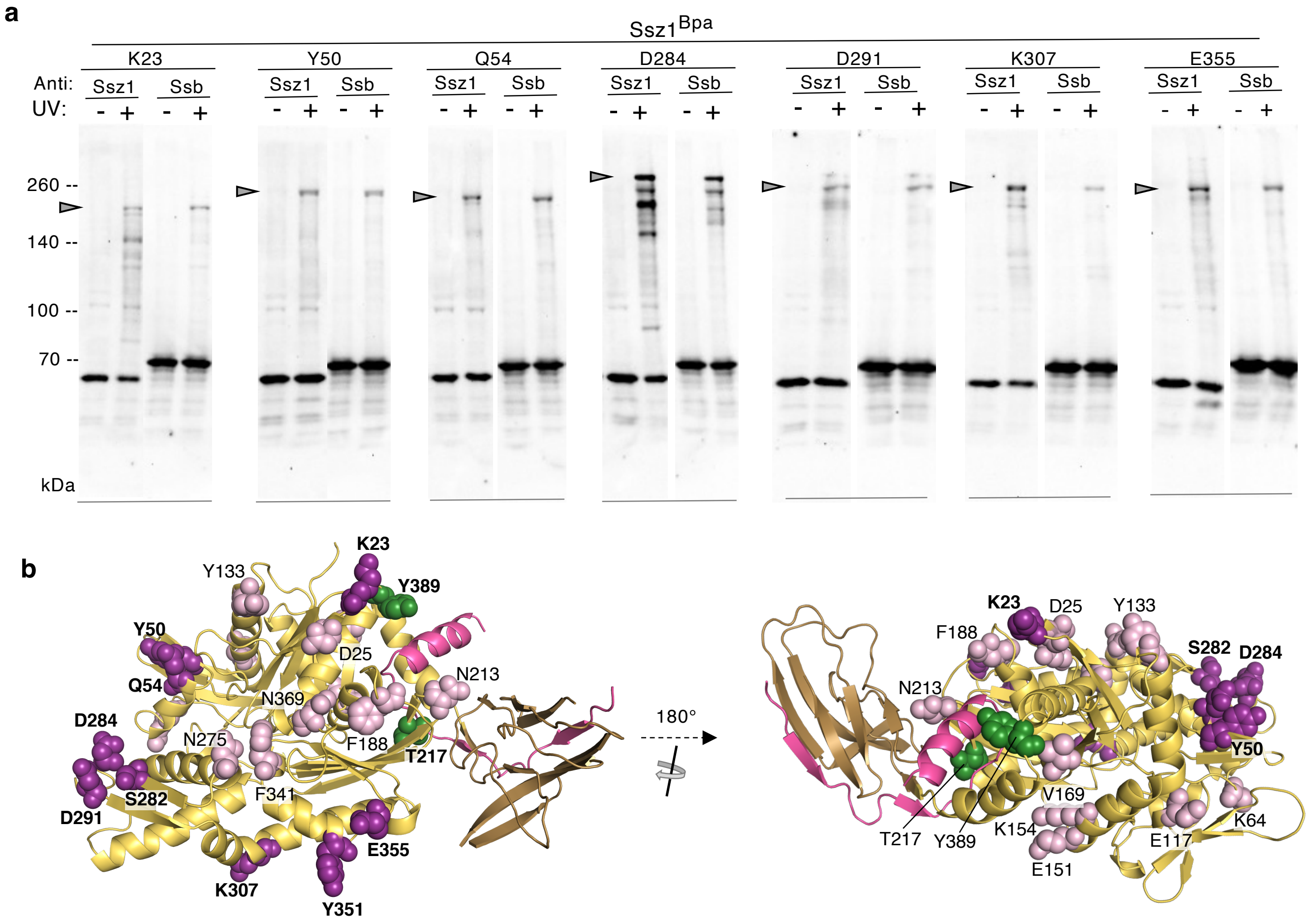

Supplementary Figure 1.
Crosslinking of Ssz1 Bpa variants.
(**a**) Second round of in vivo crosslinking of Ssz1^{Bpa} variants to Ssb. Cells expressing variants of Ssz1 with Bpa incorporated at the indicated positions were exposed to UV light (+) or left unexposed (-). Crosslinking was analyzed by immunoblotting after SDS-PAGE with antibodies specific for (anti) Ssz1 or Ssb. Ssz1^{Bpa}-Ssb crosslink products indicated by arrowhead. Migration of molecular weight markers (kDa) indicated with dash. Uncropped blots are provided as a Source Data file. Three independent strains were analyzed for each Bpa variant, with similar results.
(**b**) Model of *S. cerevisiae* Ssz1, based on PDB 5MB9 [https://www.rcsb.org/structure/5MB9]. Positions in sphere representation that yielded crosslinks to Ssb (purple) or were negative (light pink); variants that crosslinked to Zuo1 (dark green). Ssz1 NBD, beige; Ssz1 SBD, brown. Zuo1 residues 22-56 in pink.

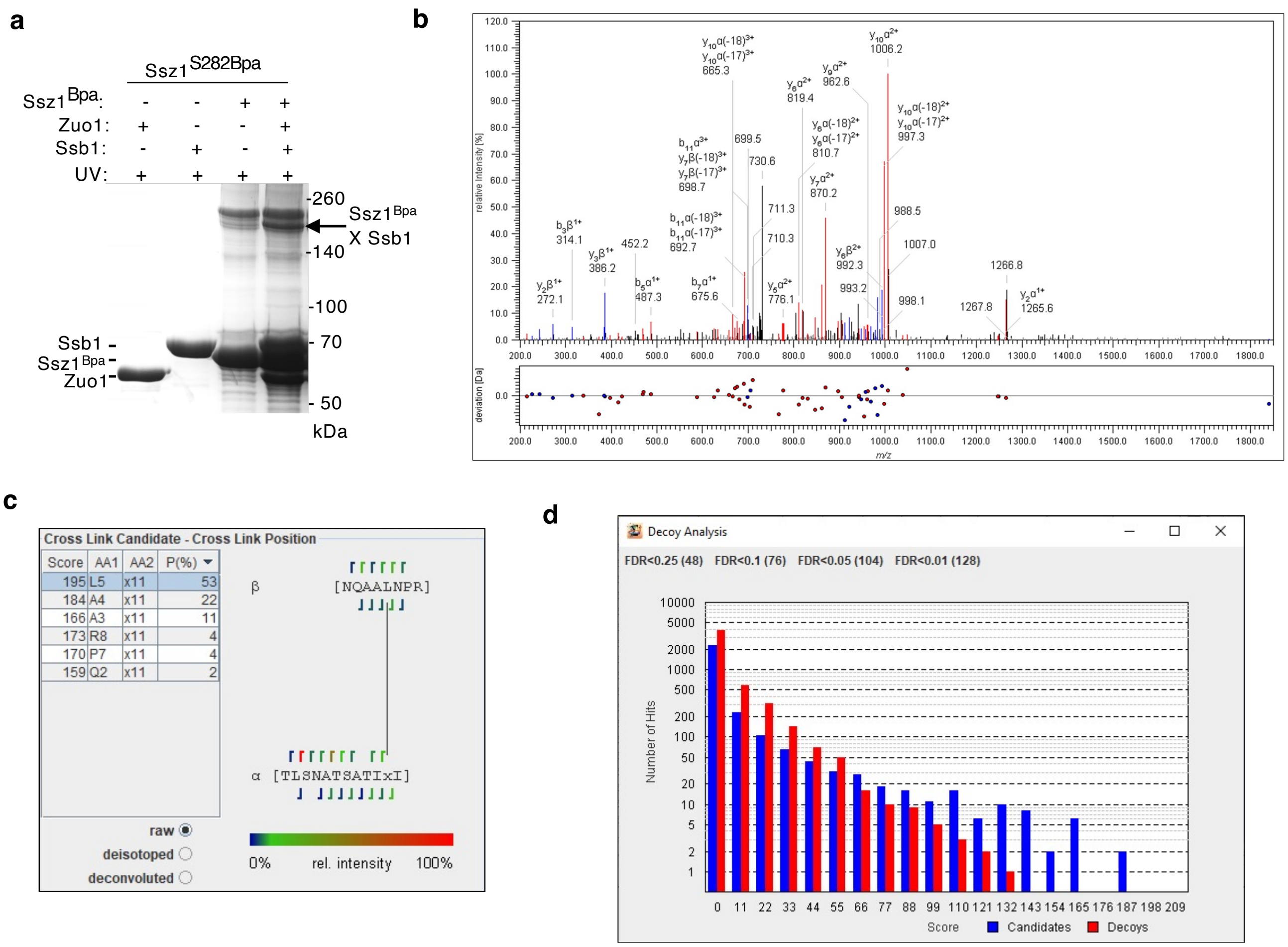

Supplementary Figure 2.

Analysis of crosslinking of Ssz1 variant having Bpa at position 282 to Ssb1 by mass spectrometry.

(**a**) Indicated combinations of purified Ssz1^{S282Bpa}, Ssb1 and Zuo1 were mixed prior to exposure to UV and then subjected to SDS-PAGE. Gel fragments containing the indicated crosslinking products between Ssz1^{S282Bpa} and Ssb1 (arrow) were excised and digested with protease for mass spectrometry analysis. Ssz1, Ssb1 and Zuo1 bands are indicated with dashes. Similar patterns of crosslinking were observed with Ssz1^{S282Bpa} obtained from three independent transformants.

(**b**) MS/MS of top crosslinking candidate of Ssz1^{S282Bpa} and Ssb1 dipeptide, α-peptide (Ssz1) ions in red and β-peptide (Ssb1) ions in blue, mass deviation of fragment ions underneath MS2 spectrum.

(**c**) Sequence of the crosslinked dipeptide and StavroX calculated probabilities for Bpa-crosslinked residues.

(**d**) StavroX global decoy analysis results.

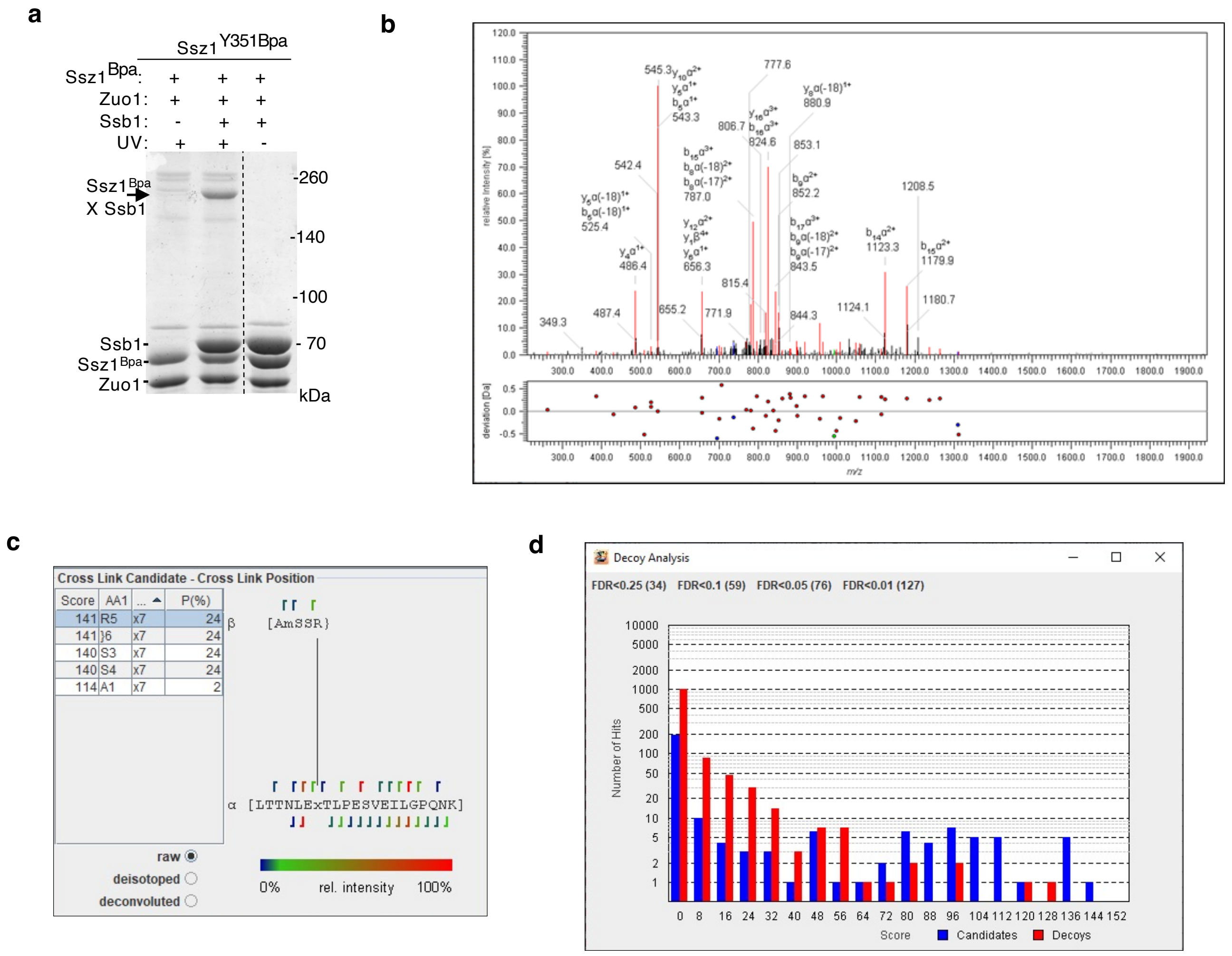

Supplementary Figure 3.

Analysis of crosslinking of Ssz1 variant having Bpa at position 351 to Ssb1 by mass spectrometry.

(**a**) Indicated combinations of purified Ssz1^{Y351Bpa}, Ssb1 and Zuo1 were mixed prior to exposure to UV (+) or left unexposed (-) and then subjected to SDS-PAGE. Gel fragments containing the indicated crosslinking products between Ssz1^{Y351Bpa} and Ssb1 (arrow) were excised and digested with protease for mass spectrometry analysis. Ssz1, Ssb1 and Zuo1 bands are indicated with dashes. The vertical dashed line indicates where nonrelevant lanes of the gel were spliced out. Similar patterns of crosslinking were observed with Ssz1^{Y351Bpa} obtained from three independent transformants.

(**b**) MS/MS of top crosslinking candidate of Ssz1^{Y351Bpa} and Ssb1 dipeptide with complete cleavage of both peptides, α-peptide (Ssz1) ions in red and β-peptide (Ssb1) ions in blue, mass deviation of fragment ions underneath MS2 spectrum.

(**c**) Sequence of the crosslinked dipeptide and StavroX calculated probabilities for Bpa-crosslinked residues. Bracket (}) indicates the C-terminus of the Ssb1 polypeptide.

(**d**) StavroX global decoy analysis results.

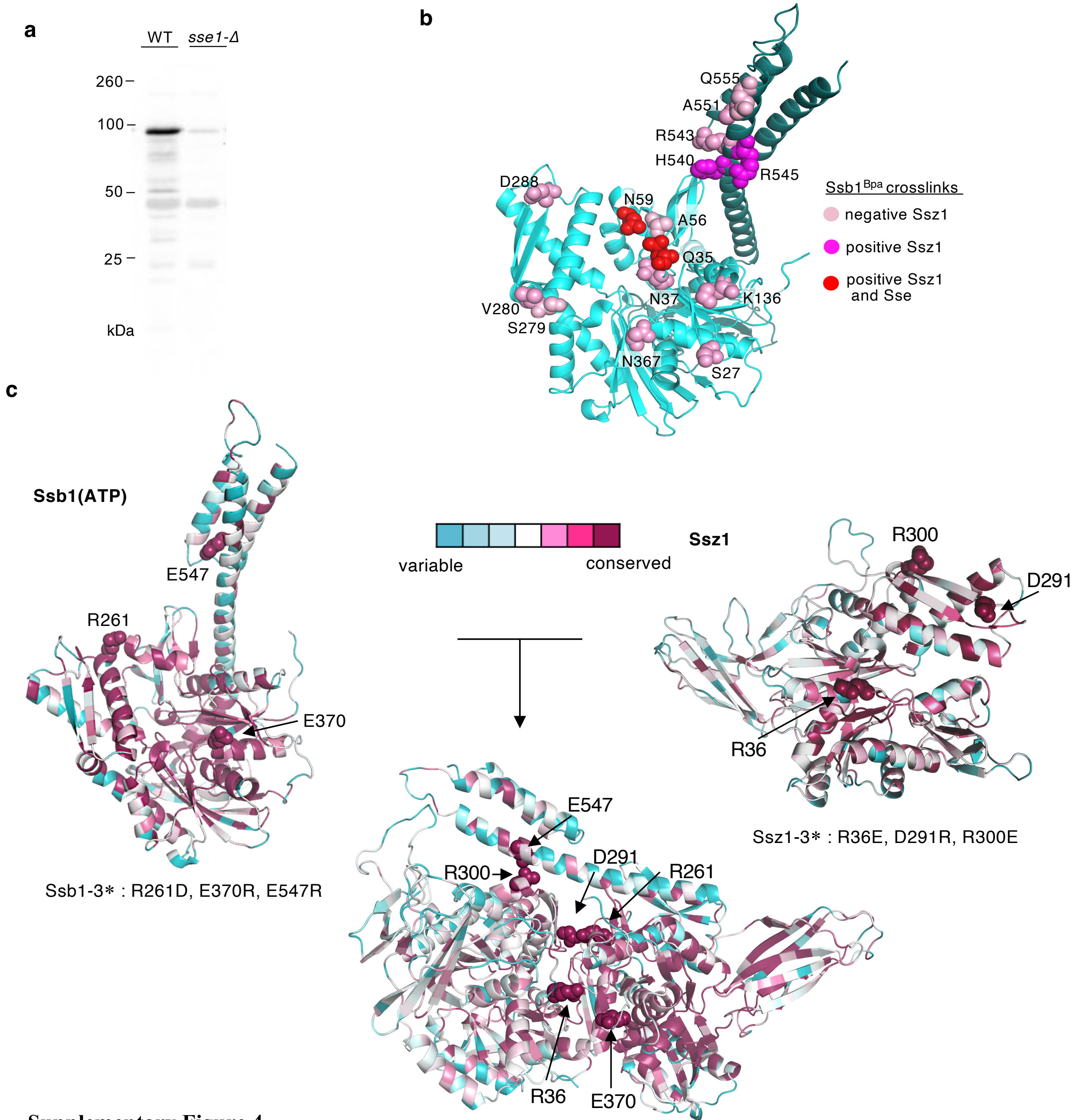

Supplementary Figure 4.
Summary of crosslinking of Ssb1^{Bpa} variants.

(**a**) Validation of Sse1 antibody directed against full-length Sse1 fused at the C-terminus to glutathione-S-transferase. Extracts of WT and *sse1*-Δ cells were subjected to electrophoresis and immunoblot analysis. Both WT and *sse1*-Δ cells express a paralog, Sse2, at a lower level than Sse1. Uncropped blots are provided as a Source Data file. Similar results were observed with three independent strains.

(**b**) Summary of results of in vivo crosslinking of Ssb1^{BPA} variants to Ssz1. Model of *S. cerevisiae* Ssb1(ATP) conformation modeled from the *C. thermophilum* Ssb1 (PDB 5TKY [https://www.rcsb.org/structure/5TKY]) crystal structure (NBD, light teal; SBD, dark teal) with positions of Bpa incorporation highlighted in sphere representation. Crosslinking: Ssz1 only (magenta), Ssz1 and Sse (red), Ssz1 negatives (light pink).

(c) Conservation of Ssz1 and Ssb1. Fungal Ssz1 or Ssb1 protein sequences were aligned and represented in the *S. cerevisiae* Ssz1 and Ssb1 structures modeled using *C. thermophilum* Ssz1 (PDB 5MB9 [https://www.rcsb.org/structure/5MB9]) and Ssb1 (PDB 5TKY [https://www.rcsb.org/structure/5TKY]) crystal structures according to conservation using the CONSURF server. The positions in the Ssz1-Ssb1 interface that were changed by mutagenesis to encode residues of opposite charge to generate the 3* variants are shown in sphere representation.

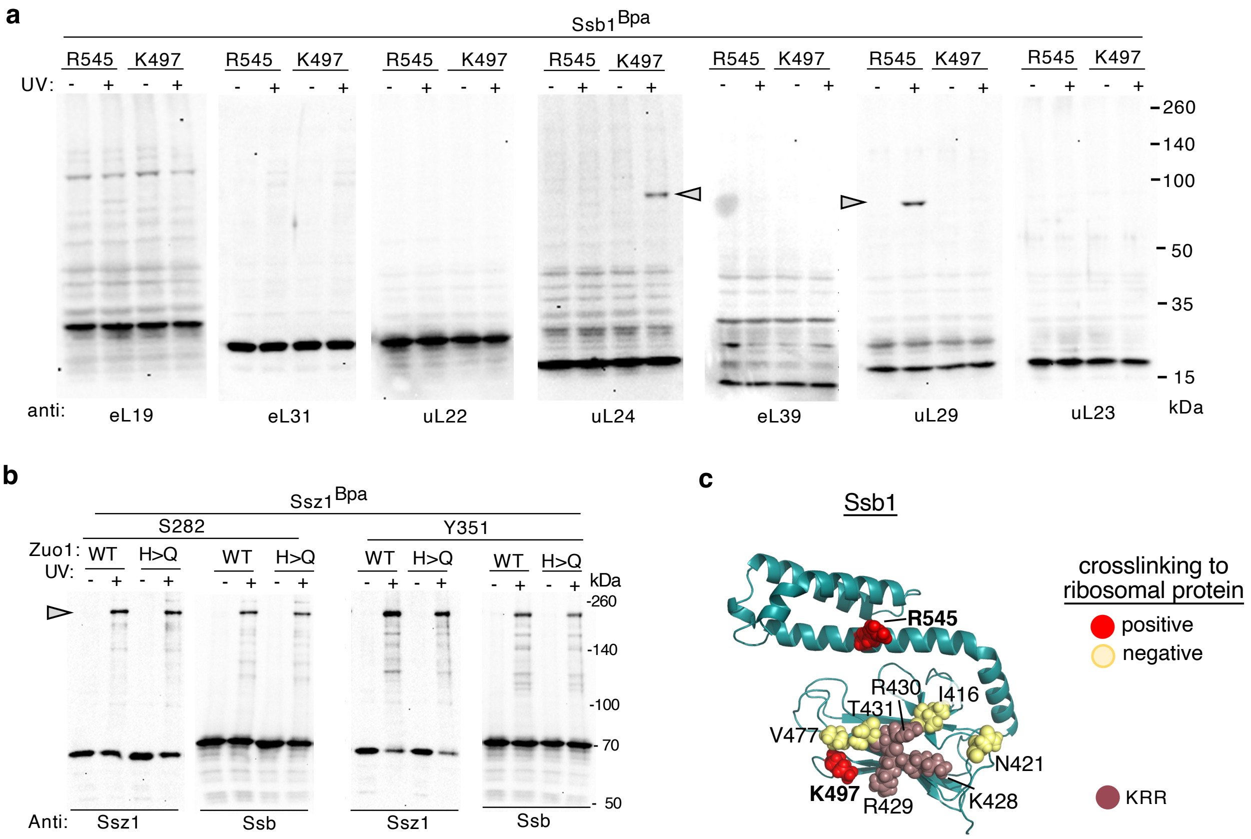

Supplementary Figure 5.

Crosslinking of Ssz1 and Ssb1 Bpa variants.

(**a**) Assessment of crosslinking of Ssb1^{R545Bpa} and Ssb1^{K497Bpa} to ribosomal proteins near the ribosomal tunnel exit. Cells expressing indicated Ssb1^{Bpa} variants were exposed to UV light (+) or left unexposed (-). Crosslinking was analyzed by immunoblotting after SDS-PAGE through a 7.5-17.5 % gradient gel with antibodies specific for indicated ribosomal proteins, with the exception of eL31, which was detected using antibody specific for the HA tag present at eL31's C-terminus. Ssb1^{Bpa}-ribosomal protein crosslink products indicated by arrowhead. Migration of molecular weight markers (MW) indicated with dashes. Uncropped blots are provided as a Source Data file. Three independent strains were analyzed for each Bpa variant, with similar results.

(**b**) Crosslinking of Ssz1^{Bpa} in *ZUO1* (WT) and *zuo1^{H128Q}* (H>Q). Cells expressing Ssz1^{S282Bpa} or Ssz1^{Y351Bpa} were exposed to UV light (+) or left unexposed (-). Crosslinking was analyzed by immunoblotting after SDS-PAGE with antibodies specific for (anti) Ssz1 or Ssb. Ssz1^{Bpa}-Ssb crosslink products indicated by arrowhead. Migration of molecular weight markers (kDa) indicated with dash. Uncropped blots are provided as a Source Data file. Three independent strains were analyzed for each Bpa variant, with similar results.

(**c**) SBD of Ssb1 when ADP is bound to the NBD modeled using the DnaK SBD structure (PDB 2KHO [https://www.rcsb.org/structure/2KHO]). The positions of SBDβ in which Bpa was incorporated and tested for Ssb crosslinking are shown in sphere representation. Negative, yellow; positive position (K497) in red, as is SBDα position K545, for reference; KRR (residues 429-430) in brown.

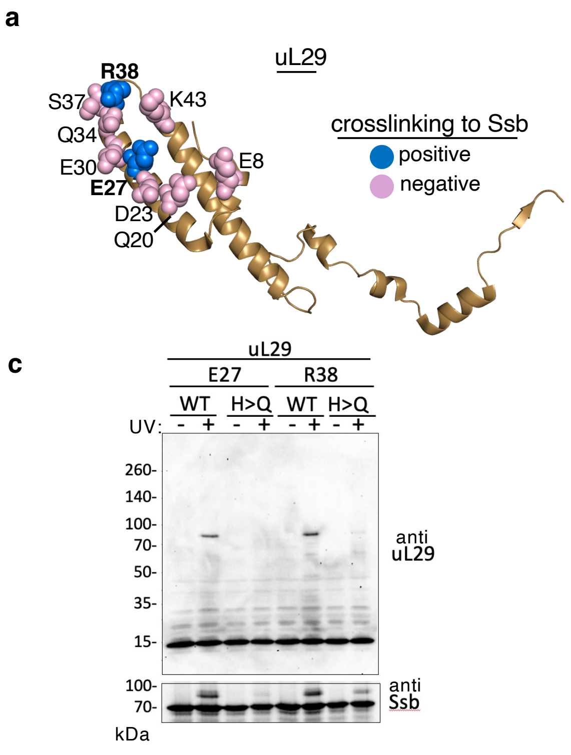

Supplementary Figure 6.
Crosslinking of uL29 and uL24 to Ssb.
(**a,b**) Positions in uL29 (a) and uL24 (b) in which Bpa was incorporated and tested for crosslinking to Ssb are shown in sphere representation. Positives in uL29 (blue) and uL24 (dark gray); negatives (light pink).
(**c,d**) *ZUO1* (WT) and *zuo1*$_{H128Q}$ (H>Q) cells expressing uL29 (c) or uL24 (d) variants with Bpa at the indicated positions were exposed to UV light (+) or, as a control, left unexposed (-). Crosslinking was analyzed by immunoblotting after SDS-PAGE in (c) a 7.5-17.5 % gradient gel or (d) or 4-20% gradient gel with antibodies specific for proteins indicated under each panel. Uncropped blots are provided as a Source Data file. Three independent strains were analyzed for each Bpa variant, with similar results.

Supplementary Tables

Supplementary Table 1. Yeast plasmids used in this study

Plasmid	Description	References
pRS316-SSB1	pRS316 carrying *SSB1* under control of its native promoter; used as template to generate all Ssb1 variants used in this study unless specified otherwise	James P et al (1997) Science **275**:387
pRS315-SSZ1TGA	pRS315 carrying *SSZ1* under control of its native promoter and with substitution of TGA stop codon in place of native TAG; used as template to generate all Ssz1TAG variants used in this study unless specified otherwise	this study
pRS314-SSZ1	pRS314 carrying *SSZ1* under control of its native promoter; used as template to generate charge-reversal mutant	Eisenman H & Craig EA (2004) Mol Microbiology **53**:335
pRS313-SSZ1	pRS313 carrying *SSZ1* under control of its native promoter and with substitution of TGA stop codon in place of native TAG; used as template to generate all Ssz1 TAG variants to be studied in the presence of Ssb1 variants	this study
pRS316-ZUO1	pRS316 carrying *ZUO1* under control of its native promoter	Yan W et al (1998) EMBO J **17**: 4809
pRS316-zuo1H128Q	pRS316 carrying $zuo1^{H128Q}$ (HPD loss of function mutant) under control of its native promoter	this study
pRS317-ZUO1	pRS317 carrying *ZUO1* under control of its native promoter	Lee K et al (2016) Nat Struct Mol Biol **23**:1003
pRS317-zuo1H128Q	pRS317 carrying $zuo1^{H128Q}$ (HPD loss of function mutant) under control of its native promoter	this study
pRS316-uL24b	pRS316 carrying the gene encoding uL24b (RPL26B) under control of its native promoter; used as template to generate all uL24b variants used in this study unless specified otherwise	this study
pRS316-uL29a	pRS316 carrying the gene encoding uL29a (RPL35A) under control of its native promoter; used as template to generate all uL29a variants used in this study unless specified otherwise	this study
pRS416-TEF-RPL31A-HA	pRS416 carrying RPL31A gene with HA-tag coding sequence at its 3'-end, under control of TEF1 promoter	Lee K et al (2016) Nat Struct Mol Biol **23**:1003
ptRNA-Bpa	2 micron plasmid encoding a variant tRNA synthetase and tRNACUA for Bpa incorporation; *TRP1* marker	Krishnamurthy M (2011) ACS Chem Biol **6**:1321

Supplementary Table 2. Yeast strains used in this study

Strain[a]	Genotype	Figure	Reference
Y1011	*Δssb1::HIS3 Δssb2::LEU2*	2a, S5a	Pfund C et al (2001) Mol Biol Cell **12**:3773
Y1216	*Δzuo1::HIS3 Δssz1::LYS2*	S5b	Hundley H et al (2002) Proc Natl Acad Sci **99**:4203
Y1218	*Δssz1::LYS2*	1b, S1a	Hundley H et al (2002) Proc Natl Acad Sci **99**:4203
Y1430	*Δssz1::LYS2 Δssb1::HIS3 Δssb2::LEU2*	3b	Hundley H et al (2002) Proc Natl Acad Sci **99**:4203
Y1437	*Δzuo1::HIS3 Δssb1::HIS3 Δssb2::LEU2*	4a, 4b	Hundley H et al (2002) Proc Natl Acad Sci **99**:4203
Y2828	*Δssz1::LYS2 Δssb1::KanMX Δssb2::LEU2*	3a	this study
Y2862	*ΔuL24b::HIS3 Δzuo1::KanMX*	4c (left), S6b	this study
Y2880	*ΔuL29a::KanMX Δzuo1::HIS3*	4c (right), S6a	this study

[a]all are DS10 strain background - *GAL2 his3-11,15 leu2-3,112 lys1 lys2 Δtrp1 ura3-52*

Supplementary Table 3. Oligonucleotides Used in Study

Oligo name	Sequence
**Ssz1 Bpa**	
Ssz1 K23TAG	ATT GCC TAC ATC AAC CCA TAG AAC GAT GTT GAT GTC ATT G
Ssz1 D25TAG	C TAC ATC AAC CCA AAG AAC TAG GTT GAT GTC ATT GCC AAC
Ssz1 Y50TAG	C TAT GTC GGT GAA GAT GAA TAG CAC GGT GGT CAA GCT TT
Ssz1 Q54TAG	GAT GAA TAC CAC GGT GGT TAG GCT TTG CAA CAA TTA ATC AG
Ssz1 K64TAG	G CAA CAA TTA ATC AGA AAT CCT TAG AAT ACT ATC ATT AAC TTC CG
Ssz1 E117TAG	G GAA GAA AAA CTT ACT GTA GAT TAG GTG GTC TCC AGA CAT TTA AAC AG
Ssz1 Y133TAG	AG TTA GCC GCG GAA GAT TAG ATC GGT TCT GCC GTA AAG
Ssz1 E151TAG	A GTT CCA ACA AAC TTC AGT TAG GAA CAA AAG ACT GCA CTA A
Ssz1 K154TAG	CA AAC TTC AGT GAA GAA CAA TAG ACT GCA CTA AAG GCT TCT
Ssz1 V169TAG	CC AAA ATT GGT CTG CAA ATT TAG CAA TTC ATC AAT GAA CCT TC
Ssz1 F188TAG	CAC GCT GAA CAA TTC CCA TAG GAA AAA GAT GTT AAC GTT GT
Ssz1 N213TAG	CT GTC ATT GCC GTT CGT TAG GGT ATT TTC ACT ATT TTG GC
Ssz1 T217TAG	C GTT CGT AAC GGT ATT TTC TAG ATT TTG GCC ACT GCT CAT
Ssz1 N275TAG	ACC AAG AAG ACT TTG TCC TAG GCA ACT TCT GCC ACT ATT
Ssz1 S282TAG	GCA ACT TCT GCC ACT ATT TAG ATC GAT TCC TTA GCT GAT G
Ssz1 D284TAG	TCT GCC ACT ATT TCC ATC TAG TCC TTA GCT GAT GGT TTC
Ssz1 D291TAG	TCC TTA GCT GAT GGT TTC TAG TAT CAC GCT TCT ATC AAC A
Ssz1 K307TAG	AGG TAC GAA TTG GTA GCT AAC TAG GTT CGC CCA ATT TTC
Ssz1 F341TAG	TTG ACT GGT GGT GTA TCA TAG ACT CCA AAA TTA ACC ACT AA
Ssz1 Y351TAG	CA AAA TTA ACC ACT AAC TTG GAA TAG ACT TTA CCA GAA TCA GTC GAA ATT C
Ssz1 E355TAG	CT AAC TTG GAA TAC ACT TTA CCA TAG TCA GTC GAA ATT CTT GGT CC
Ssz1 N369TAG	CAG AAC AAG AAC GCT TCT TAG AAT CCA AAC GAA TTA GCT G
Ssz1 Y389TAG	A GCA AGA TTG ATT AGC GAT TAG GAT GCT GAC GAA TTG GC
**Ssz1 3* mutant**	
Ssz1 R36E	CC AAC CCA GAT GGT GAG GAA GCC ATT CCA TCC GCT TT
Ssz1 D291R	TCC TTA GCT GAT GGT TTC CGT TAT CAC GCT TCT ATC AAC A

Supplementary Table 3. Oligonucleotides Used in Study

Ssz1 R300E	C GCT TCT ATC AAC AGA ATG GAA TAC GAA TTG GTA GCT AAC A
**Ssb1 3* mutant**	
Ssb1 R261D	C ATC TCC GAC GAT GCC GAT GCT TTG AGA AGA TTG AGA A
Ssb1 E370R	GAA AAA TCT ATT AAC CCA GAT CGT GCT GTT GCT TAC GGT GC
Ssb1 E547R	C GAA GCT AGA CAA AGA TTG CGT TCC TAC GTT GCC TCC AT
**Ssb1 Bpa**	
Ssb1 S27TAG	GTT GCT ACT TAC GAA TCC TAG GTT GAA ATT ATT GCC AAC G
Ssb1 Q35TAG	TT GAA ATT ATT GCC AAC GAA TAG GGT AAC AGA GTC ACC CC
Ssb1 N37TAG	ATT GCC AAC GAA CAA GGT TAG AGA GTC ACC CCA TCT TTC
Ssb1 A56TAG	AA GAA AGA TTG ATT GGT GAT TAG GCC AAG AAC CAA GCT GC
Ssb1 N59TAG	TT GGT GAT GCT GCC AAG TAG CAA GCT GCT TTG AAC CC
Ssb1 K136TAG	G AAG GAA ATT GCT GAA GCT TAG ATT GGT AAG AAG GTT GAA AA
Ssb1 S279TAG	A GCT AAG AGA ACC TTA TCT TAG GTC ACT CAA ACT ACC GTT
Ssb1 V280TAG	CT AAG AGA ACC TTA TCT TCT TAG ACT CAA ACT ACC GTT GAA G
Ssb1 D288TAG	T CAA ACT ACC GTT GAA GTT TAG TCT TTG TTT GAC GGT GAA G
Ssb1 N367TAG	AAG CAA TTG GAA AAA TCT ATT TAG CCA GAT GAA GCT GTT GC
Ssb1 I416TAG	CAA GGT GAC ATG TTC GGT TAG GTT GTT CCA AGA AAC ACT ACT G
Ssb1 N421TAG	TC GGT ATC GTT GTT CCA AGA TAG ACT ACT GTT CCA ACC ATC AAG
Ssb1 T431TAG	GTT CCA ACC ATC AAG AGA AGA TAG TTT ACT ACA TGT GCT GAC AAC
Ssb1 V477TAG	G ATG CCA GCT GGT GAA CCA TAG TTG GAA GCT ATC TTC GAA G
Ssb1 K497TAG	TG AAG GTT ACT GCC GTC GAA TAG TCT ACC GGT AAG TCC TCT AAC
Ssb1 H540TAG	GAA GCT TTT GCC AAG AAG TAG GAA GCT AGA CAA AGA TTG G
Ssb1 R543TAG	GCC AAG AAG CAC GAA GCT TAG CAA AGA TTG GAA TCC TAC G
Ssb1 R545TAG	C AAG AAG CAC GAA GCT AGA CAA TAG TTG GAA TCC TAC GTT GCC TC
Ssb1 A551TAG	CAA AGA TTG GAA TCC TAC GTT TAG TCC ATC GAA CAA ACT GTC
Ssb1 Q555TAG	C GTT GCC TCC ATC GAA TAG ACT GTC ACT GAC CCA GTC
**uL24 Bpa**	
uL24 K37TAG	G TTA TCT GCT CCA TTA TCC TAG GAA TTG AGA GCT CAA TAT G

Supplementary Table 3. Oligonucleotides Used in Study

uL24 Q42TAG	CC AAG GAA TTG AGA GCT TAG TAT GGT ATC AAG GCT TTG CC
uL24 L57TAG	C AGA AGA GAC GAT GAA GTC TAG GTT GTT CGT GGT TCC AAG
uL24 K64TAG	G GTT GTT CGT GGT TCC AAG TAG GGT CAA GAA GGT AAG ATT TC
uL24 K84TAG	G TTT GCT GTT CAA GTT GAC TAG GTC ACC AAG GAA AAG GTC
uL24 E88TAG	GTT GAC AAG GTC ACC AAG TAG AAG GTC AAC GGT GCT TCC G
**uL29Bpa**	
uL29 E8TAG	G GCC GGT GTT AAA GCT TAC TAG CTA AGA ACC AAA TCC AAG G
uL29 Q20TAG	C AAG GAA CAA TTG GCT TCT TAG TTG GTT GAC TTG AAA AAG G
uL29 D23TAG	CAA TTG GCT TCT CAA TTG GTT TAG TTG AAA AAG GAG TTG GCT G
uL29 E27TAG	CAA TTG GTT GAC TTG AAA AAG TAG TTG GCT GAA TTG AAG GTC C
uL29 E30TAG	C TTG AAA AAG GAG TTG GCT TAG TTG AAG GTC CAA AAG TTG TC
uL29 Q34TAG	G TTG GCT GAA TTG AAG GTC TAG AAG TTA TCC AGA CCA TCT TTG
uL29 S37TAG	GAA TTG AAG GTC CAA AAG TTG TAG AGA CCA TCT TTG CCA AAG
uL29 R38TAG	G AAG GTC CAA AAG TTG TCC TAG CCA TCT TTG CCA AAG ATC AAG
uL29 K43TAG	CC AGA CCA TCT TTG CCA TAG ATC AAG ACC GTC AGA AAG AG
**Gene isolation**	
uL24b5pri*	GGCCGCTCTAGAACTAGTGCATGTCCAGGTTCGAAGCTG
uL24b3pri*	GAATTCCTGCAGCCCGGGGAGCCCTTGAGACCCGAAGGC
uL29a5pri*	GGCCGCTCTAGAACTAGTGACTGTTCAAGACTGAAGAGG
uL29a3pri*	GAATTCCTGCAGCCCGGGGTTGACCCTAATCAAATAAGG

*All oligonucleotides were also synthesized as reverse complement to facilitate quick change mutagenesis –with the exception of those marked with * used for gene isolation.
