# Supplementary material for: Pathway of Hsp70 interactions at the ribosome
Source: Nat Commun. 2021 Sep 27;12:5666. doi: 10.1038/s41467-021-25930-8 (PMC8476630; doi:10.1038/s41467-021-25930-8)
Supplement: Supplementary file 3 — Reporting Summary [file 41467_2021_25930_MOESM3_ESM.pdf]

nature research

Corresponding author(s): Elizabeth A Craig

Last updated by author(s): Sep 2, 2021

Reporting Summary

Nature Research wishes to improve the reproducibility of the work that we publish. This form provides structure for consistency and transparency in reporting. For further information on Nature Research policies, see our Editorial Policies and the Editorial Policy Checklist.

Statistics

For all statistical analyses, confirm that the following items are present in the figure legend, table legend, main text, or Methods section.

n/a	Confirmed	
☐	☒	The exact sample size (*n*) for each experimental group/condition, given as a discrete number and unit of measurement
☐	☒	A statement on whether measurements were taken from distinct samples or whether the same sample was measured repeatedly
☒	☐	The statistical test(s) used AND whether they are one- or two-sided<br>*Only common tests should be described solely by name; describe more complex techniques in the Methods section.*
☒	☐	A description of all covariates tested
☒	☐	A description of any assumptions or corrections, such as tests of normality and adjustment for multiple comparisons
☒	☐	A full description of the statistical parameters including central tendency (e.g. means) or other basic estimates (e.g. regression coefficient) AND variation (e.g. standard deviation) or associated estimates of uncertainty (e.g. confidence intervals)
☒	☐	For null hypothesis testing, the test statistic (e.g. *F*, *t*, *r*) with confidence intervals, effect sizes, degrees of freedom and *P* value noted<br>*Give P values as exact values whenever suitable.*
☒	☐	For Bayesian analysis, information on the choice of priors and Markov chain Monte Carlo settings
☒	☐	For hierarchical and complex designs, identification of the appropriate level for tests and full reporting of outcomes
☒	☐	Estimates of effect sizes (e.g. Cohen's *d*, Pearson's *r*), indicating how they were calculated

Our web collection on statistics for biologists contains articles on many of the points above.

Software and code

Policy information about availability of computer code

Data collection	All blot images were acquired with an iBright CL1000 (Invitrogen) imaging system running version 1.6.0 software with automatic exposure settings.<br>Peptides were analyzed by nanoLC-MS/MS using the Agilent 1100 nanoflow system (Agilent) connected to a hybrid linear ion trap-orbitrap mass spectrometer (LTQ-Orbitrap Elite™, Thermo Fisher Scientific) equipped with an EASY-Spray™ electrospray source.
Data analysis	PyMOL 2.4 and 2.5.0 Schrodinger, LLC<br>ConSurf server: https://consurf.tau.ac.il/<br>Modeller 9.23<br>StavroX Freeware version 3.6.6. from University of Halle-Wittenberg<br>MSConvert 3.0 (ProteoWizard: Open Source Software for Rapid Proteomics Tools Development)<br>Mascot search engine 2.2.07 (Matrix Science)<br>ZDOCK server: https://zdock.umassmed.edu/<br>ImageJ 1.53a

For manuscripts utilizing custom algorithms or software that are central to the research but not yet described in published literature, software must be made available to editors and reviewers. We strongly encourage code deposition in a community repository (e.g. GitHub). See the Nature Research guidelines for submitting code & software for further information.

Data

Policy information about availability of data

All manuscripts must include a data availability statement. This statement should provide the following information, where applicable:

- Accession codes, unique identifiers, or web links for publicly available datasets
- A list of figures that have associated raw data
- A description of any restrictions on data availability

The mass spectrometry proteomics data have been deposited to the ProteomeXchange Consortium via the PRIDE63 partner repository with the dataset identifier PXD024065 (http://www.ebi.ac.uk/pride/archive/projects/PXD024065). Other data that support the findings of this study and biological materials are available from the corresponding authors on reasonable request. Source data are provided with this paper for immunoblots. That Saccharomyces Genome database [https://www.yeastgenome.org] was used to derive gene sequences. Structural information was derived from the RSCB Protein Data Base (PDB) [http://rcsb.org]: PDB 5MB9 [https://www.rcsb.org/structure/5MB9]; PDB 5TKY [https://www.rcsb.org/structure/5TKY]; PDB 3D2F [https://www.rcsb.org/structure/3D2F]; PDB 3GL1 [https://www.rcsb.org/structure/3GL1]; PDB 2KHO [https://www.rcsb.org/structure/2KHO]; PDB 3J78 [https://www.rcsb.org/structure/3J78]; PDB 5DJE [https://www.rcsb.org/structure/5DJE]; PDB 5NRO [https://www.rcsb.org/structure/5NRO]

Field-specific reporting

Please select the one below that is the best fit for your research. If you are not sure, read the appropriate sections before making your selection.

☒ Life sciences ☐ Behavioural & social sciences ☐ Ecological, evolutionary & environmental sciences

For a reference copy of the document with all sections, see nature.com/documents/nr-reporting-summary-flat.pdf

Life sciences study design

All studies must disclose on these points even when the disclosure is negative.

Sample size	All yeast experiments were conducted at least in triplicate (biological replicates, that is independent yeast transformants). For MS/MS n=1 for each Bpa variant, with multiple individual peptides identified within the sample.
Data exclusions	No data was excluded.
Replication	Replications was done using independent yeast transformants.
Randomization	Experiments were carried out with randomly selected yeast transformants from different cultures having the same genetic background.
Blinding	Experiments were performed comparing various samples having various treatments; it was necessary for the researchers to be aware of treatment such as transformation or temperature. Appropriate cellular and genetic controls were included in each replication.

Reporting for specific materials, systems and methods

We require information from authors about some types of materials, experimental systems and methods used in many studies. Here, indicate whether each material, system or method listed is relevant to your study. If you are not sure if a list item applies to your research, read the appropriate section before selecting a response.

Materials & experimental systems

n/a	Involved in the study	
☐	☒	Antibodies
☒	☐	Eukaryotic cell lines
☒	☐	Palaeontology and archaeology
☒	☐	Animals and other organisms
☒	☐	Human research participants
☒	☐	Clinical data
☒	☐	Dual use research of concern

Methods

n/a	Involved in the study	
☒	☐	ChIP-seq
☒	☐	Flow cytometry
☒	☐	MRI-based neuroimaging

Antibodies

Antibodies used	All antibodies used were rabbit polyclonals. Antibodies specific for ribosomal proteins eL19, uL22, uL23, uL24, uL29 and uL39 were kindly provided by Sabine Rospert. Antibody for HA tag was obtained from Proteintech Group, Inc. (cat.# 51064-2-AP). Secondary antibody was from Amersham (cat.# NA934-1ML) - ECL anti-rabbit, HRP-linked. Antibodies for Ssa, Ssb, Ssz1 and Zuo1 were made "in house".

Validation

Antibodies specific for ribosomal proteins eL19, uL22, uL23, uL24, uL29 and uL39 were kindly provided by the Rospert lab (PMID 177119726, 24072706). The Ssb (PMID 9860955), Ssz1 (PM15225326) and Zuo1 (PMID 27669034) antibodies were reported and validated previously. Validation of Sse antibodies is reported here by testing a strain lacking the Sse1 gene (see Supplementary Fig 4a).
